# Global Melanoma Correlations With Obesity, Smoking, and Alcohol Consumption

**DOI:** 10.2196/31275

**Published:** 2021-12-13

**Authors:** Nisha Batta, Sarah Shangraw, Andrew Nicklawsky, Takeshi Yamauchi, Zili Zhai, Dinoop Ravindran Menon, Dexiang Gao, Robert P Dellavalle, Mayumi Fujita

**Affiliations:** 1 Department of Dermatology University of Colorado Anschutz Medical Campus Aurora, CO United States; 2 Department of Biostatistics and Informatics University of Colorado Anschutz Medical Campus Aurora, CO United States; 3 Rocky Mountain Regional Veterans Affairs Medical Center Aurora, CO United States; 4 Department of Epidemiology Colorado School of Public Health University of Colorado Anschutz Medical Campus Aurora, CO United States; 5 Department of Immunology and Microbiology University of Colorado Anschutz Medical Campus Aurora, CO United States

**Keywords:** melanoma incidence, melanoma mortality, non-UV risk factors, obesity, alcohol consumption, smoking, wine, World Health Organization, WHO, Global Cancer Observatory, GCO, Global Health Observatory, GHO, aldehyde dehydrogenase 2, ALDH2, polymorphism

The incidence of melanoma has continued to rise over the last few decades [1]. Although many explanations have been posited, such as increased screening, detection, and UV exposure [2], it is essential to examine non-UV–related risk factors contributing to its continued rise.

We reviewed published data on obesity, smoking, and alcohol consumption trends worldwide to understand human behaviors and their relationship to melanoma. We collected data for the three risk factors from the Global Health Observatory (GHO), World Health Organization (WHO), published in 2010, as these were the most comprehensive available data with minimal changes in trends noted in the following years. We also collected data for melanoma incidence and mortality from the Global Cancer Observatory (GCO), WHO, published in 2018, as they were the most currently available data. Compiled data were displayed using choropleth maps with color gradients to visualize variations across geographic areas (Figure 1A). Subsequently, each country’s data were plotted, and Spearman rank correlation coefficient (R) was calculated for melanoma incidence and mortality with each risk factor. The choropleth map of each risk factor showed similar patterns to melanoma incidence and melanoma mortality. The statistical analysis depicted a positive correlation (with a positive R) between melanoma incidence/mortality and all risk factors (obesity, smoking, and alcohol consumption). Among them, alcohol consumption showed the strongest positive correlation with both melanoma incidence (*R*=0.72; *P*<.001) and mortality (*R*=0.59; *P*<.001). Because individuals with lighter skin color (eg, Caucasians) have a higher melanoma incidence, this correlation data might implicate that alcohol consumption is high in countries with lighter skin color, such as European ancestry. To address whether the correlation between alcohol consumption and melanoma incidence is skin color dependent or independent, we reanalyzed the data by continent (Figure 1B). A positive correlation still existed between alcohol consumption and melanoma incidence in Europe, Asia, and Africa (Multimedia Appendix 1, Supplementary Table S1). In particular, the strongest correlation (*R*=0.68; *P*<.001) was observed in European countries with exclusively lighter skin color (1-12 or 12-14 of skin color numbers, per a human skin color distribution map in the second figure by Barsh [3]), suggesting that the correlation between alcohol consumption and melanoma incidence is likely to be skin color independent. A positive correlation was also observed between alcohol consumption and melanoma mortality in all continents.

To understand how genetic risk factors have a role in the observed correlation between alcohol consumption and melanoma incidence/mortality, we examined the correlation of aldehyde dehydrogenase 2 (ALDH2) rs671 polymorphism with both alcohol consumption and melanoma outcomes. ALDH2 is an alcohol-metabolizing enzyme, and its allelic variation affects alcohol detoxification [4]. The correlation analysis showed that it was the wild-type ALDH2 allele that was strongly positively correlated with melanoma incidence (*R*=0.70; *P*<.001) and mortality (*R*=0.74; *P*<.001; Table 1). On the other hand, the allelic variants had a modest to strong negative correlation with melanoma incidence (*R*=–0.70 to –0.51; *P*<.001) and mortality (*R*=–0.73 to –0.57; *P*<.001). Possible explanations for the opposing effect of ALDH2 polymorphism include that individuals with risk alleles consume less alcohol than wild-type individuals, which is consistent with our data showing a positive and a negative correlation to alcohol consumption in wild-type individuals and allelic variants, respectively.

Overall, our findings highlight an association between alcohol and melanoma outcomes globally. The association was observed not only with melanoma incidence but also with its mortality. We also found a potential involvement of the alcohol-related gene ALDH2. Limitations of our analyses include unavailability of the population statistics for some risk factors by some countries, binary questionnaire of alcohol use without reflecting the quantity of alcohol consumption, and country-based analysis rather than individualized data. To determine whether our cross-country data support individual-level conclusions at individual levels, individual-level studies, such as a recent one [5], need to be conducted. Furthermore, our findings do not necessarily indicate causation from alcohol, and other factors might be involved, including skin/hair color, ethnicity, geography, economy, and lifestyle. Further investigation is warranted to verify these associations at individual levels and elucidate alcohol’s effects on melanoma outcomes by eliminating potential confounding factors such as skin/hair color genotypes.

**Figure 1:**
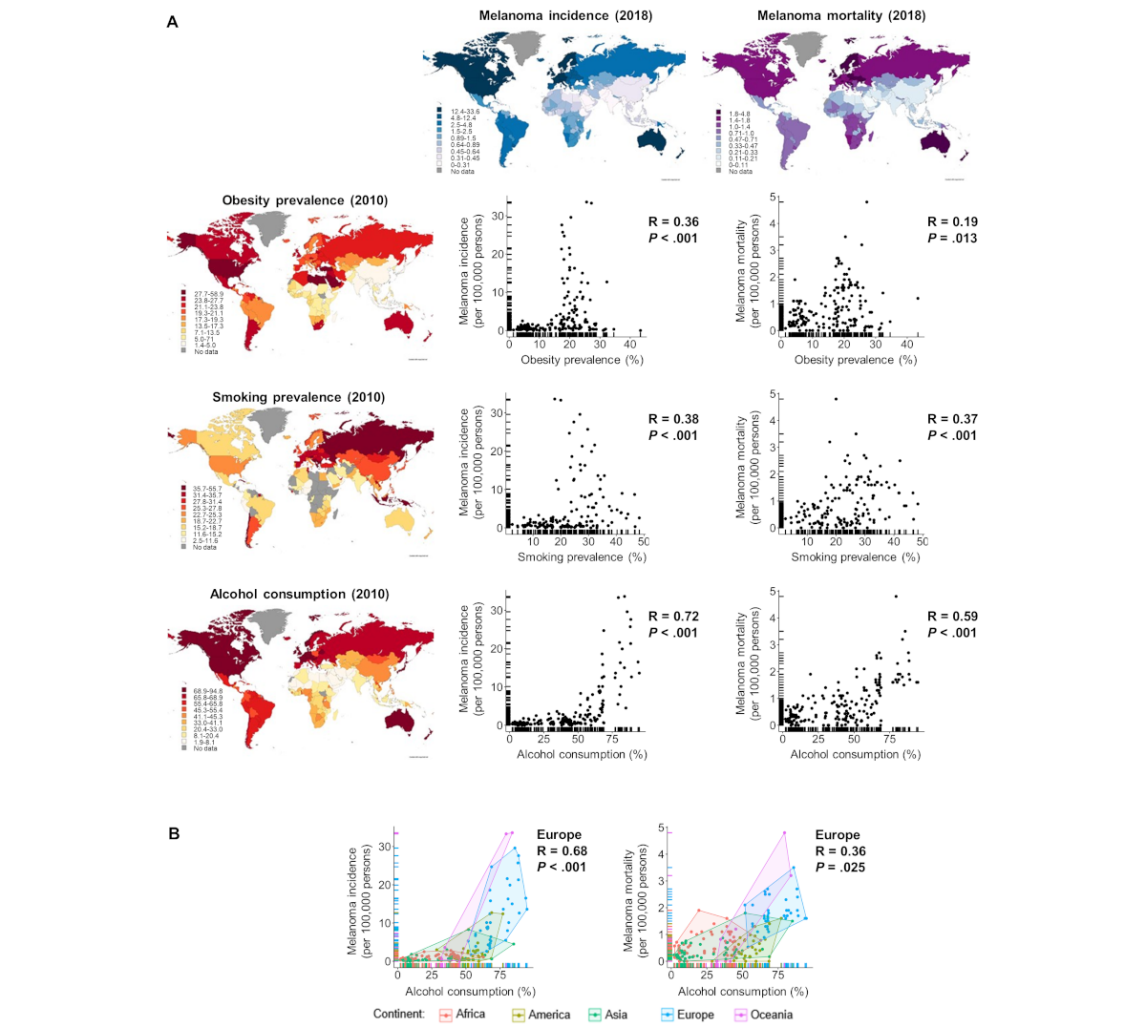
Global maps and scatter plots of melanoma burden and potential lifestyle factors. A: Correlation of melanoma outcomes and 3 lifestyle factors. Melanoma incidence (2018) is the number of new melanoma cases in 2018, including both sexes and all ages, and expressed as a rate per 100,000 persons per year. Melanoma mortality (2018) is the number of deaths due to melanoma in 2018, including both sexes and all ages, and expressed as a rate per 100,000 persons per year. Obesity prevalence (2010) refers to the percentage of obesity among adults (20+ years, both sexes) with a BMI of 30 kg/m2 or higher in 2010. Smoking prevalence (2010) refers to the percentage of men and women ages 15 and older who smoked any tobacco product daily or nondaily in 2010. Smokeless tobacco use is excluded and not available from the original data source. Alcohol consumption (2010) refers to the proportion of adults (15+ years, both sexes) who consumed any alcohol in 2010. Sources: Global Cancer Observatory (melanoma incidence and mortality) and Global Health Observatory (obesity prevalence, smoking prevalence, and alcohol consumption) from World Health Organization. The choropleth maps were created with MapChart. Scatter plots of each country’s metrics were created to visualize the distributions of lifestyle factors and outcomes. The left panel of scatter plots: correlation of melanoma incidence with obesity prevalence (n=174), smoking prevalence (n=140), and alcohol consumption (n=175). The right panel of scatter plots: correlation of melanoma mortality with obesity prevalence (n=174), smoking prevalence (n=140), and alcohol consumption (n=175). The original data set included the following number of countries: melanoma incidence and mortality (n=185), obesity prevalence (n=174), smoking prevalence (n=140), and alcohol consumption (n=190). B: Correlation of alcohol consumption and melanoma outcome by continent. Figure 1A data were reanalyzed by grouping countries into continents: Africa (n=55), Americas (n=39), Asia (n=48), Europe (n=40), and Oceania (n=17; Multimedia Appendix 1, Supplementary Table S1). Each symbol represents an individual country. Hashes on each axis are included to assist in visualizing the distribution of each variable. Spearman rank coefficient was used to assess correlation due to skewed data and the influence of outliers. *P* values were reported based on a null hypothesis of no monotonic association against a two-sided alternative at the .05 level. The statistical analysis was conducted using R version 4.0.2.

**Table 1:** Correlations between melanoma outcomes and ALDH2 alleles in 23 countries.^a^

Variable		Correlation with					
		Melanoma incidence	*P* value	Melanoma mortality	*P* value	Alcohol consumption	*P* value
**Genetic alleles**							
	ALDH2^b^ *1/*1	0.70	<.001	0.74	<.001	0.39	.07
	ALDH2 *1/*2	–0.70	<.001	–0.73	<.001	–0.38	.07
	ALDH2 *2/*2	–0.51	.01	–0.57	.005	–0.25	.26
Alcohol consumption		0.79	<.001	0.71	<.001	N/A^c^	N/A

^a^The source of melanoma incidence (2018), melanoma mortality (2018), and alcohol consumption (2010) is explained in the Figure 1 legend. ALDH2 allele frequency was obtained by searching research papers (Multimedia Appendix 1, Supplementary Table S2). The original data set included the following number of countries: melanoma incidence (n=185), melanoma mortality (n=185), alcohol consumption (n=175), and ALDH2 alleles (n=23). Only 23 countries had all 4 factors available for the correlation analysis. Spearman rank coefficient was used to assess correlation due to skewed data and the influence of outliers. The data represent correlation coefficients (R) with *P* values. Alcohol consumption was reassessed to determine the correlation coefficient with an associated *P* value for the subset of countries that were considered. The statistical analysis was conducted using R version 4.0.2 (R Foundation for Statistical Computing).

^b^ALDH2: aldehyde dehydrogenase 2.

^c^N/A: not applicable.

## Appendix

Multimedia Appendix 1 Supplemental tables.

## Abbreviations

ALDH2: aldehyde dehydrogenase 2
WHO: World Health Organization
