# Supplementary material for: Global Melanoma Correlations With Obesity, Smoking, and Alcohol Consumption
Source: JMIR Dermatol. 2021 Dec 13;4(2):e31275. doi: 10.2196/31275 (PMC9387903; doi:10.2196/31275)
Supplement: Multimedia Appendix 1 [file derma_v4i2e31275_app1.pptx]

## Slide 1
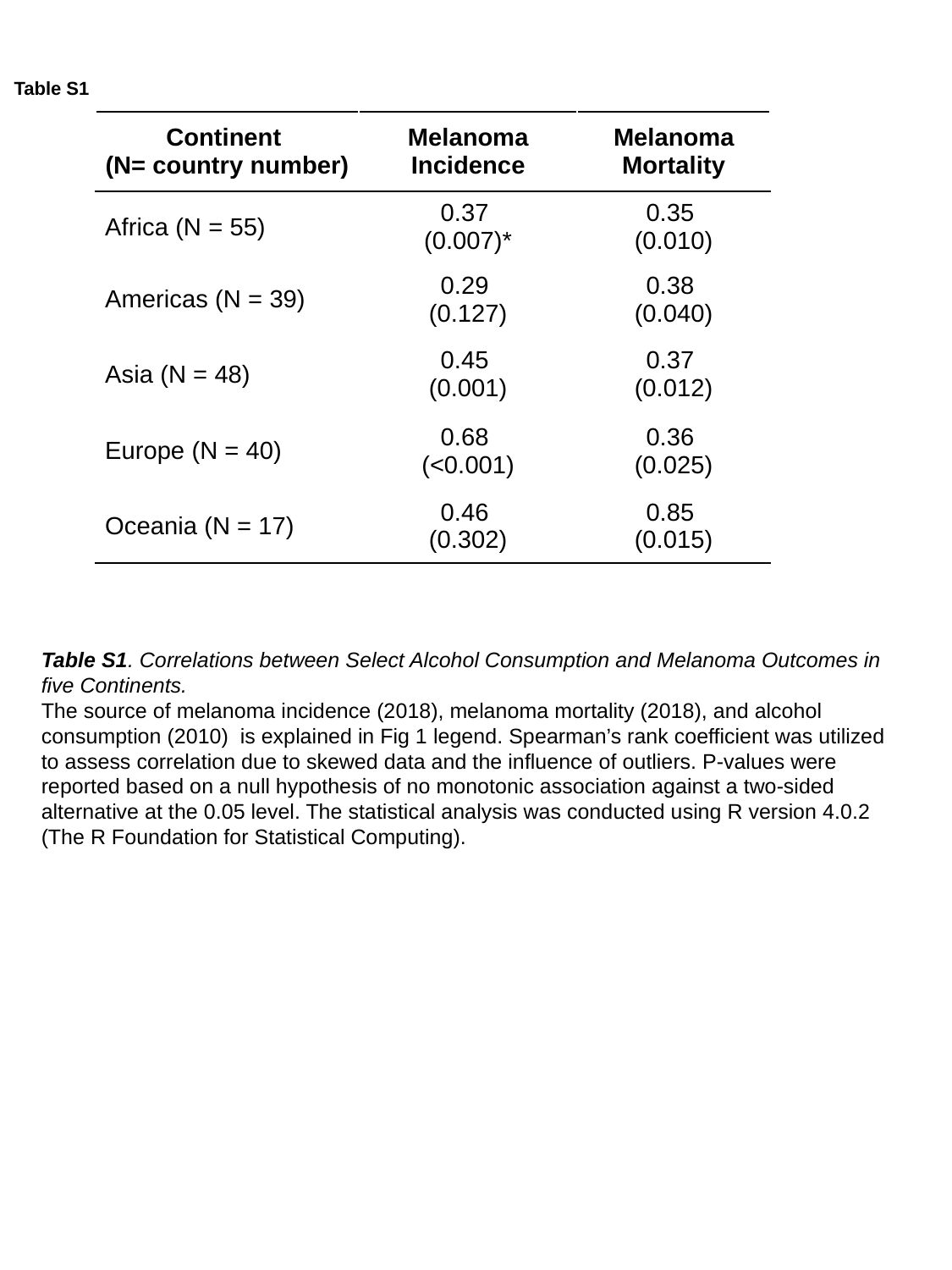

Table S1
| Continent (N= country number) | Melanoma Incidence | Melanoma Mortality |
| --- | --- | --- |
| Africa (N = 55) | 0.37 (0.007)\* | 0.35 (0.010) |
| Americas (N = 39) | 0.29 (0.127) | 0.38 (0.040) |
| Asia (N = 48) | 0.45 (0.001) | 0.37 (0.012) |
| Europe (N = 40) | 0.68 (<0.001) | 0.36 (0.025) |
| Oceania (N = 17) | 0.46 (0.302) | 0.85 (0.015) |
Table S1. Correlations between Select Alcohol Consumption and Melanoma Outcomes in five Continents.
The source of melanoma incidence (2018), melanoma mortality (2018), and alcohol consumption (2010) is explained in Fig 1 legend. Spearman’s rank coefficient was utilized to assess correlation due to skewed data and the influence of outliers. P-values were reported based on a null hypothesis of no monotonic association against a two-sided alternative at the 0.05 level. The statistical analysis was conducted using R version 4.0.2 (The R Foundation for Statistical Computing).

## Slide 2
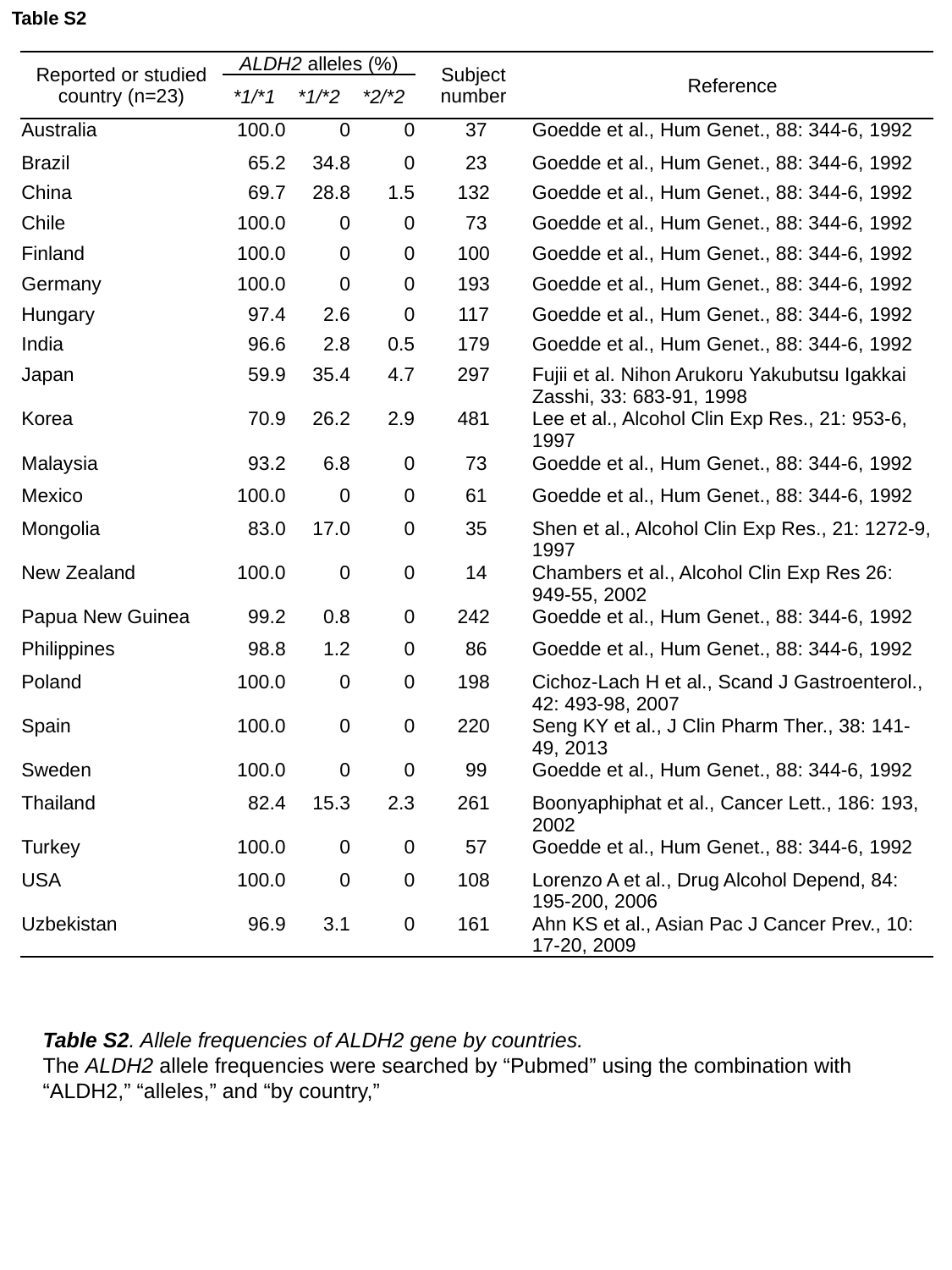

Table S2
| Reported or studied country (n=23) | ALDH2 alleles (%) | | | Subject number | Reference |
| --- | --- | --- | --- | --- | --- |
| | \*1/\*1 | \*1/\*2 | \*2/\*2 | | |
| Australia | 100.0 | 0 | 0 | 37 | Goedde et al., Hum Genet., 88: 344-6, 1992 |
| Brazil | 65.2 | 34.8 | 0 | 23 | Goedde et al., Hum Genet., 88: 344-6, 1992 |
| China | 69.7 | 28.8 | 1.5 | 132 | Goedde et al., Hum Genet., 88: 344-6, 1992 |
| Chile | 100.0 | 0 | 0 | 73 | Goedde et al., Hum Genet., 88: 344-6, 1992 |
| Finland | 100.0 | 0 | 0 | 100 | Goedde et al., Hum Genet., 88: 344-6, 1992 |
| Germany | 100.0 | 0 | 0 | 193 | Goedde et al., Hum Genet., 88: 344-6, 1992 |
| Hungary | 97.4 | 2.6 | 0 | 117 | Goedde et al., Hum Genet., 88: 344-6, 1992 |
| India | 96.6 | 2.8 | 0.5 | 179 | Goedde et al., Hum Genet., 88: 344-6, 1992 |
| Japan | 59.9 | 35.4 | 4.7 | 297 | Fujii et al. Nihon Arukoru Yakubutsu Igakkai Zasshi, 33: 683-91, 1998 |
| Korea | 70.9 | 26.2 | 2.9 | 481 | Lee et al., Alcohol Clin Exp Res., 21: 953-6, 1997 |
| Malaysia | 93.2 | 6.8 | 0 | 73 | Goedde et al., Hum Genet., 88: 344-6, 1992 |
| Mexico | 100.0 | 0 | 0 | 61 | Goedde et al., Hum Genet., 88: 344-6, 1992 |
| Mongolia | 83.0 | 17.0 | 0 | 35 | Shen et al., Alcohol Clin Exp Res., 21: 1272-9, 1997 |
| New Zealand | 100.0 | 0 | 0 | 14 | Chambers et al., Alcohol Clin Exp Res 26: 949-55, 2002 |
| Papua New Guinea | 99.2 | 0.8 | 0 | 242 | Goedde et al., Hum Genet., 88: 344-6, 1992 |
| Philippines | 98.8 | 1.2 | 0 | 86 | Goedde et al., Hum Genet., 88: 344-6, 1992 |
| Poland | 100.0 | 0 | 0 | 198 | Cichoz-Lach H et al., Scand J Gastroenterol., 42: 493-98, 2007 |
| Spain | 100.0 | 0 | 0 | 220 | Seng KY et al., J Clin Pharm Ther., 38: 141-49, 2013 |
| Sweden | 100.0 | 0 | 0 | 99 | Goedde et al., Hum Genet., 88: 344-6, 1992 |
| Thailand | 82.4 | 15.3 | 2.3 | 261 | Boonyaphiphat et al., Cancer Lett., 186: 193, 2002 |
| Turkey | 100.0 | 0 | 0 | 57 | Goedde et al., Hum Genet., 88: 344-6, 1992 |
| USA | 100.0 | 0 | 0 | 108 | Lorenzo A et al., Drug Alcohol Depend, 84: 195-200, 2006 |
| Uzbekistan | 96.9 | 3.1 | 0 | 161 | Ahn KS et al., Asian Pac J Cancer Prev., 10: 17-20, 2009 |
Table S2. Allele frequencies of ALDH2 gene by countries.
The ALDH2 allele frequencies were searched by “Pubmed” using the combination with “ALDH2,” “alleles,” and “by country,”
